# Mary Crosse project: systematic reviews and grading the value of neonatal tests in predicting long term outcomes

**DOI:** 10.1186/1471-2393-9-49

**Published:** 2009-10-29

**Authors:** Gemma L Malin, Rachel K Morris, Khalid S Khan

**Affiliations:** 1 Academic Department of Obstetrics and Gynaecology, School of Clinical and Experimental Medicine, University of Birmingham, Birmingham Women's Hospital, Birmingham. B15 2TG. UK

## Abstract

**Background:**

Events before birth, condition at birth, events immediately following birth, and condition in early childhood are linked together, and have implications for health and disease in adulthood. At present, there is lack of clarity about the tests that purport to link these various stages. This is partly because there is paucity of collated information about the best strategies for predicting longer-term outcomes before (using tests in fetal period) or after birth (using tests in neonatal period, infancy as well as early childhood).

**Methods/Design:**

A series of systematic reviews and meta-analyses will be undertaken to determine, amongst neonates, the ability of various tests and measures to predict infant, childhood and adult outcomes. We will search Medline, Embase, Cochrane Library, MEDION, citation lists of review articles and eligible primary articles and will contact experts in the field. Independent reviewers will select studies, extract data and assess study quality according to established criteria. Language restrictions will not be applied. Data synthesis will involve meta-analysis (where appropriate), exploration of heterogeneity and publication bias. Evidence collated will be graded for its quality to support decision making.

**Discussion:**

The project will collate, synthesise and evaluate the available evidence concerning the value of tests of neonatal wellbeing to predict long term outcomes. The systematic reviews will assess the quality of available evidence and identify tests with the strongest association with outcomes, and assess their economic value. The output of this project will help formulate practice recommendations.

## Background

Events before birth, condition at birth, events immediately following birth, and condition in early childhood are linked together, and may have implications for health and disease in adulthood [1]. A variety of parameters are used to assess neonatal wellbeing such as the APGAR score,[2] umbilical cord pH, [3] need for neonatal intensive care and growth measurements including birth weight, head circumference and skin fold thickness [4]. Studies of tests or interventions in pregnancy and labour often use these factors as outcome measures [5]. Similarly, complications in childhood such as cerebral palsy may be attributed to antenatal or intrapartum events where there is an abnormal neonatal test such as low cord pH or low birth weight. However, there are conflicting results in existing studies regarding the strength of association between an abnormal neonatal test and adverse outcomes. A comprehensive systematic review of the literature on all available tests can improve our ability to identify those infants at greatest risk of developing immediate, childhood and adult complications.

Let us for example, take umbilical cord pH at birth, defined as the pH and base excess in arterial and venous samples, measured from a segment of umbilical cord which is double clamped immediately after delivery. It is widely used as an objective measure of perinatal asphyxia, a major cause of neonatal and childhood morbidity and mortality worldwide. Acidaemia at birth has been associated with neonatal complications such as hypoxic ischaemic encephalopathy and seizures, [6] liver dysfunction,[7] acute renal impairment,[8] death,[9] and long term morbidity such as cerebral palsy and developmental delay [10]. Pathological fetal acidosis is considered to occur at an arterial cord pH of <7.00 and a base deficit ≥ 12 mmol/l, levels to which cerebral palsy is often attributed [10]. The criteria have been derived through consensus statement rather than through evaluation of collated evidence summaries in this field [11,12]. Existing studies of the association between pH levels and outcomes have drawn inconsistent inferences. This discrepancy may be due to the different parameters measured (arterial or venous pH and base excess), the different thresholds used to define abnormality, and the variety of outcomes evaluated. This and other inconsistencies in the literature on neonatal testing will be explored in our review.

The APGAR score, too, has been widely used for many years to quantify the neonatal condition at birth, considering heart rate, respiratory effort, colour and tone at 1, 5 and 9 minutes of age. Although it provides a useful summary of an infant's condition, studies correlating it to long term outcomes have varied widely in their findings [4,13]. The significance of a low APGAR score where the clinical condition improves quickly is therefore uncertain, and will be investigated within the scope of this project. Similarly, measures at birth for fetal growth restriction have been associated with neonatal mortality,[14] childhood disability and impaired neurodevelopment,[15,16] educational disadvantage and disease in adult life (e.g. diabetes mellitus, hypertension) [17,18]. However, a variety of different reference criteria for confirmation of growth restriction are used, including absolute birth weight <2500 g, birth weight < 10^th ^centile adjusted for gestational age and local population values, and neonatal ponderal index < 10^th ^centile [19]. There is lack of consensus as to which of these reference standards and thresholds has the strongest correlation with adverse outcome. This review will consider each parameter in turn and assess the association of neonatal, childhood and adult outcomes with each.

The need for admission to neonatal intensive care (NICU) is widely used as a reference standard for overall neonatal morbidity. However, the policy for admitting neonates varies widely between intensive care units both nationally and internationally, for example some would admit all babies born to diabetic mothers for a period of observation [20]. This variation may affect the association of NICU admission with long term outcomes. More specific assessment of initial neonatal morbidity involves scoring systems used in the intensive care setting such as the Clinical Risk Index for Babies (CRIB) and the Score for Neonatal Acute Physiology (SNAP). We will assess the correlation between these scores and short and long term outcomes.

Clarification of the correlation between neonatal tests and subsequent outcomes is necessary to optimise clinical decision making and counselling of parents when an infant is affected. In turn, a better understanding of the long term associations of neonatal tests will improve understanding of the implications of tests and interventions in pregnancy that affect neonatal outcomes.

## Methods/Design

Funded by the Mary Crosse Fund at Birmingham Women's Hospital a systematic review project based on this protocol will be conducted.

In 1973 Dr Crosse bequeathed the legacy of her estate to the former South Birmingham Hospital Management Committee for the development of research in Maternity, Neonatal and Special Care Baby Unit.

### Objectives

To determine the association and clinical impact of neonatal findings and tests (including birth weight, Apgar scores and umbilical cord pH) with morbidity and mortality in infancy, childhood and adulthood, using systematic reviews and meta-analyses.

### Search Strategy

Literature will be identified using:

• General bibliographic databases including MEDLINE (PubMED) and EMBASE (OVID)

• Specialist electronic databases: the Cochrane Library (DARE, CCTR), MEDION

• Contact with individual experts and those with an interest in this field to uncover grey literature

• Hand- searching of selected specialist journals

• Checking of reference lists of relevant review articles and papers that will be eligible for inclusion

Searches will we performed to identify the neonatal tests in question and combined with a search to identify morbidity and mortality. The comprehensive search strategy will aim to find all primary studies reporting the association of each neonatal test with any measure of childhood or adult morbidity and mortality. The search strategy for umbilical cord pH may be viewed as an additional file 1 (other searches are available for authors on request). Search terms related to the test (e.g. Umbilical cord, Hydrogen-ion concentration, Asphyxia neonatorum, umbilical artery pH, cord pH) are combined using 'and' with MESH headings (e.g. Human development, Infant mortality) and keywords (e.g. developmental delay, handicap) to encompass neonatal mortality and short and long term morbidity. The search will be restricted to human studies only. No language restrictions will be applied. All databases will be searched from inception and updated at 6 monthly intervals. A comprehensive database of the literature will be constructed (Reference Manager 11.0) to allow us to handle citations efficiently [21].

### Inclusion Criteria

Studies will be selected for inclusion in the reviews using the selection criteria based on population, index test, reference standard and study design of interest.

#### Population

Neonates in any health care setting

#### Tests

**Figure 1:**
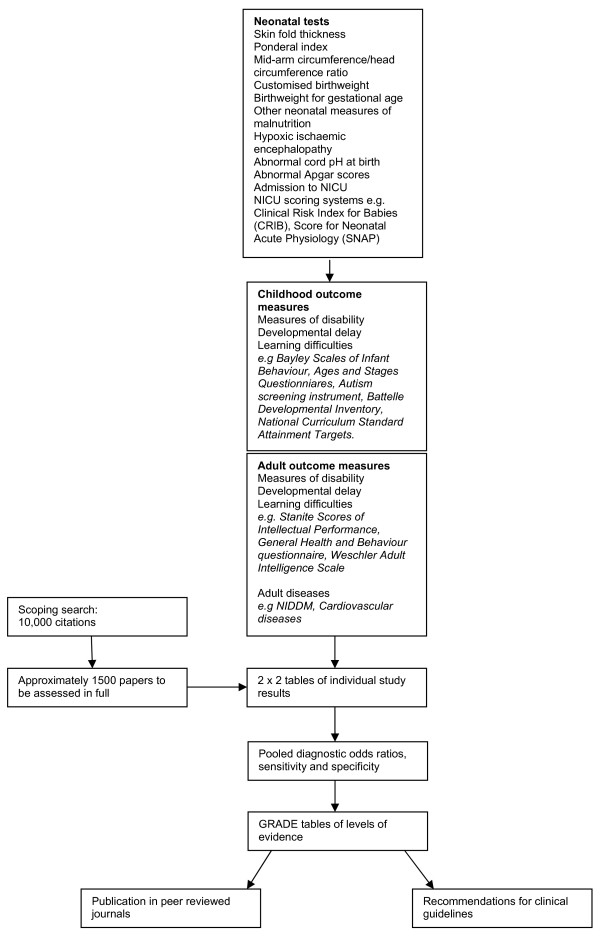
**Flow diagram to illustrate neonatal tests and outcomes to be examined in systematic reviews**.

neonatal tests will be prioritised on the basis of clinical relevance after consultation with experts in the field (figure 1).

#### Outcome measure

Any measure of infant, childhood or adult morbidity or mortality, (figure 1).

#### Study design

Observational studies (cohorts, case-control) allowing generation of 2 × 2 tables of the association between neonatal test and outcome measure. Case series ≤ 5 will be excluded due to the likely association with bias and imprecision.

##### Study selection process

Studies will be selected for inclusion in the review in a two stage process using the selection criteria detailed above. Firstly, the titles and abstracts of the citations in the Reference Manager database will be assessed by one reviewer. All papers felt to be relevant will be obtained in full text version. Two independent reviewers will then select the studies which meet predefined criteria, defined prior to commencement and individualised for each review. Disagreements will be resolved by consensus or input from a third reviewer.

##### Data Extraction

A data extraction form will be designed for each review; variations between reviews will mainly be on the information extracted regarding the index test. Data will be extracted on: identification of study (first author, year of publication, country of investigation, language of paper); population (health care setting, number of participating centres, level of risk assigned by author and clinical data on risk factors, inclusion period); study design (design, data collection, enrolment, completeness of follow up); index test (gestation, method of performing test, intra and inter-observer variation, cut off level); reference standard (incidence, reference standard used, cut off level, total number of individuals analysed for results); results (necessary data for construction of 2 × 2 table, all results will be collected for reported index tests at any cut-off level, any measure of statistical accuracy reported).

The data extraction will be conducted in duplicate using the pre-designed form. Disagreements between reviewers will again be resolved by consensus or arbitration. Where multiple publications are identified, only the most recent and/or complete study will be included. Data will be entered onto an Excel spreadsheet.

### Study quality assessment

Study and reporting quality will be assessed by at least one reviewer for all included manuscripts. Methodologic quality is a construct defined as the confidence that the study design, conduct and analysis minimises bias[22] in the estimation of the association between test and outcome, thereby maintaining internal validity (i.e. the degree to which the results of this observation are correct for the patients being studied). Another construct is that it is a set of parameters in the design and conduct of a study that reflects the validity of the outcome, related to the external and internal validity and the statistical model used [23]. For our review these parameters will be developed adapting the QUADAS tool [24]. Elements of study design which may have a direct relationship to bias and variation in a test accuracy study will be assessed with elements of the STARD checklist [25]. We have used such tools in our previous work [26].

In the assessment of study quality, prospective recruitment of patients with a consecutive or random recruitment pattern will be considered ideal. Sufficient clinical information should be given to assign a level of risk of complications, which ideally should be stated by the authors. The quality of performance and reporting of the index test will be assessed to look at elements of the test that may introduce bias. Information regarding the reference standard including method of determination, execution and blinding will be extracted. Ideal study design will be cohort studies; case control study design has been shown to affect accuracy and where numbers of studies permit these will be excluded from meta-analysis [27]. Verification bias will be assessed using a flow diagram to assess the number of eligible individuals completing both index test and outcome measure, and those excluded from the analysis with reasons. With ideal verification studies will account for all eligible individuals, state how indeterminate results were handled, and > 90% of those undergoing the index test should progress to complete the outcome measure. Where possible an individual quality assessment will be tailored to each review, using the most important items from validated tools. The assessment of quality will be represented by a stacked bar chart.

We will use the GRADE approach to determine whether we could recommend the use of each test in a clinical context. This approach is transparent in its considerations [28]. This considers the quality of the evidence not only according to the test accuracy, but the impact of the test on patient-important outcomes and takes into account factors influencing the quality of the evidence such as the study design, potential sources of bias and the precision of the results [29].

### Data description

For each test, information on individual studies will be summarised as follows:

• **Table with methodological and reporting characteristics of included studies**

The table will state the number of women in each study, the incidence of each adverse outcome (based on the number of analysed cases divided by the total number of individuals at baseline).

• **Summary of quality and reporting items of the included studies**

Results will be presented as 100% stacked bars, where the bars represent a quality item and the figures in the stacks represent the number of studies

• **Forest plots of odds ratios and 95% CIs**

Odds ratios, analysed as (true positive/false positive)/(false negative/true negative) will be presented.

• **Table with subgroup analyses **(if applicable)

• **Grade tables**

For each test the tables will state the number of studies, design, limitations, test results with outcomes important to patients, the indirectness of the impact of the test result on patient-important outcomes, the precision of the data, publication bias and an assessment of the overall quality of the evidence.

### Statistical Analyses

From the 2 × 2 tables, odds ratios will be calculated for each study along with their 95% confidence intervals (CIs) [30]. When 2 × 2 tables contain zero cells, 0.5 will be added to each cell to enable calculations [31]. In each review, results will be visualised using Forest plots and ROC plots; extreme values, outliers and threshold phenomena will be explored.

Results will be analysed in groups according to the index test performed and the outcome measure studied, these will be defined a priori for each review. Meta-analysis will be used when appropriate. Pooled summary estimates will be produced in the form of odds ratios, as these are often relatively constant regardless of the diagnostic threshold and are frequently used to demonstrate a causal association in epidemiological studies [32]. The range of uncertainty will be calculated using the 95% confidence intervals of the odds ratios for each test. A fixed or random effects model will be used as appropriate depending on the degree of heterogeneity present.

Heterogeneity of results between studies will be assessed graphically by inspection of forest plots and ROC plots. The X^2 ^and inconsistency squared will be used as statistical measures of heterogeneity. Where heterogeneity is not present (X^2 ^>0.10, p < 0.05 and I^2 ^< 50%) the fixed effect pooling method will be used and where relevant we will consider the use of the bivariate meta-regression model [22,33]. Where heterogeneity is present, this will be explored using meta-regression analyses. Factors considered to be important beforehand will be used for the analysis, including:

• Variations in population, high and low risk depending antenatal or intrapartum factors

• Study quality

• Study design: Prospective vs. Retrospective data collection

• Variations in the type of index test and outcome measure and the thresholds used

Analysis for the assessing the risk of publication bias will be carried out by producing funnel plots of accuracy estimates against corresponding variances [28]. When no publication bias is suspected the plots will be symmetrical and funnel shaped because smaller studies are expected to have increased variation in estimates of accuracy.

When interpreting the data we will consider the criteria proposed by Hill to establish causality [34]. The consistency of the results, the biological plausibility of the findings and the specificity and temporality of the associations demonstrated will be examined.

Data syntheses will be performed using meta-disc version 1.4, STATA version 10.0 and StatsDirect version 2.7.2.

## Discussion

This project will comply with guidelines on conducting systematic reviews of diagnostic tests. The methodology of diagnostic systematic reviews is rapidly evolving with a focus on assessing the effect of study design and quality on accuracy.

This project will utilise all recent developments in the methodology and statistical analysis of systematic reviews. This will include bivariate meta-analysis, a technique which analyses sensitivity and specificity jointly, accounting for the presence of a threshold effect and correlation between the two measures. We will also utilise guidelines on the methodology of systematic reviews to assess causation. The results of the review will help produce a set of neonatal tests to predict neonatal, childhood and adult morbidity and mortality, which can be used to inform clinical management of these individuals. The recently recommended GRADE approach to rating the quality of evidence and the strength of the recommendations made on the results will comprehensively explain the findings of our reviews and the rationale behind our recommendations to enable the confident use of our results to influence current practice and recommend further research.

The anticipated problems in this project include the variety of outcome measures purported to be associated with long term outcomes and the likely variety of definitions and thresholds for these outcomes. This will provide challenges to searching, and the search strategies employed will necessarily be broad, leading to a large database of potential studies to be examined. The heterogeneous nature of the outcomes may limit meta-analysis. In order to combat this problem we will perform meta-analysis according to pre-defined clinically relevant groups of outcome measures and we will explore any remaining heterogeneity with meta-regression. Our ability to establish causality may be limited by the reporting in the primary studies, for example assessment of dose-response relationships are dependent on the reporting of multiple thresholds; if the primary studies report a single cut-off then the dose-response curve would be difficult to explore. Likewise the specificity of the outcomes in relation to the test examined relies on the primary studies reporting other possible causative factors, such as the gestation at birth when examining the relationship or umbilical cord pH with cerebral palsy, as both may influence the outcome and therefore confound the results. In grading the evidence the main challenges are likely to arise from the lack of direct evidence of the impact of the test on patient outcomes. For example, there is a lack of proven interventions to improve long term outcomes in individuals with an abnormal test at birth. We will therefore have to infer benefit based on increased certainty to the patient of a normal or abnormal outcome, which will inevitably weaken the strength of our recommendations. However, areas where there is a paucity of data can be identified and used to guide future primary research. Results will be published through 2009-2011.

## Competing interests

The authors declare that they have no competing interests.

## Authors' contributions

RKM and KSK obtained the funding and with GLM developed the protocol.

All authors read and approved the final manuscript.

## Pre-publication history

The pre-publication history for this paper can be accessed here:



## Supplementary Material

Additional file 1 **Search strategy for systematic review to explore the association of umbilical cord pH at birth with neonatal and long term outcomes**. Medline search strategy employed for this systematic review. Adapted for use in other electronic databases. Click here for file
