# Supplementary material for: Mary Crosse project: systematic reviews and grading the value of neonatal tests in predicting long term outcomes
Source: BMC Pregnancy Childbirth. 2009 Oct 29;9:49. doi: 10.1186/1471-2393-9-49 (PMC2774285; doi:10.1186/1471-2393-9-49)
Supplement: Additional file 1 — Search strategy for systematic review to explore the association of umbilical cord pH at birth with neonatal and long term outcomes. Medline search strategy employed for this systematic review. Adapted for use in other electronic databases. [file 1471-2393-9-49-S1.doc]

**Additional document 1:** Medline search strategy for the association of umbilical cord pH with neonatal and long term outcomes

1. exp Infant, Newborn/

2. Asphyxia Neonatorum/

3. exp Brain Damage, Chronic/

4. exp Hypoxia-Ischemia, Brain/

5. exp mental disorders diagnosed in childhood/

6. exp Human Development/

7. Infant Mortality/

8. Child Mortality/

9. feeding difficulties.mp.

10. cord pH.mp.

11. umbilical artery pH.mp.

12. umbilical cord blood.mp.

13. exp Umbilical Cord/

14. exp Hydrogen-Ion Concentration/

15. 13 and 14

16. cerebral palsy.mp.

17. developmental delay.mp.

18. hypoxic ischaemic encephalopathy.mp.

19. handicap*.mp.

20. mental retard*.mp.

21. 10 or 11 or 12 or 13

22. exp Child/

23. 1 or 22

24. 2 or 3 or 4 or 5 or 6 or 7 or 8 or 9 or 16 or 17 or 18 or 19 or 20

25. 10 or 11 or 12 or 15

26. 24 and 25

27. cord gases.mp.

28. Blood Gas Analysis/

29. 13 and 28

30. 10 or 11 or 12 or 15 or 27 or 29

31. 24 and 30

32. Blood/ or blood.mp.

33. 13 and 32

34. 10 or 11 or 12 or 15 or 29 or 33

35. limit 34 to humans
